# Early Optimal Parenteral Nutrition During NICU Stay and Neurodevelopmental Outcomes in Very Preterm Infants: State of the Art

**DOI:** 10.3390/nu17020232

**Published:** 2025-01-09

**Authors:** Francesca Tesser, Marta Meneghelli, Diletta Martino, Luca Pegoraro, Maria Sofia Pelosi, Sofia Sebellin, Giovanna Verlato

**Affiliations:** 1 Neonatal Intensive Care Unit, Department of Women’s and Children’s Health, University Hospital of Padova, 35128 Padova, Italy; francesca.tesser@aopd.veneto.it (F.T.); marta.meneghelli@aopd.veneto.it (M.M.); diletta.martino7@gmail.com (D.M.); pegoraroluca@outlook.com (L.P.); mariasofia.pelosi@aopd.veneto.it (M.S.P.); sofia.sebellin@aopd.veneto.it (S.S.); 2 Paediatric Nutrition Service, Department of Women’s and Children’s Health, University Hospital of Padova, 35128 Padova, Italy

**Keywords:** preterm newborn, parenteral nutrition, energy, macronutrients, neurodevelopment

## Abstract

Background: Preterm infants (PIs) are more susceptible to neurodevelopmental impairment compared with term newborns. Adequate postnatal growth has been associated with improved neurocognitive outcomes; therefore, optimization of nutrition may positively impact the neurodevelopment of PIs. Objective: This study focused on macronutrient parenteral nutrition (PN) intake during the Neonatal Intensive Care Unit stay and their associations with neurodevelopmental outcomes in PIs in the first two years of life. Methods: The Embase, MEDLINE, and Cochrane Library databases were searched using the following subject headings and terms (MeSH): “premature infants”, “parenteral nutrition”, “growth”, “brain”, “neurodevelopment”, and “central nervous system diseases”. All relevant papers’ reference lists were manually searched. PN and neurodevelopment studies concerning the first two years of life were collected and analyzed. Results: 275 potential studies were retrieved, 64 were selected for full-text reading, and 22 were included (12 randomized controlled trials). While glucose intakes should be immediately provided and strictly monitored avoiding hyperglycemia, the long-term outcomes of aggressive PN caloric intakes are uncertain. Early amino acid (AA) supplementation is mandatory and improves short-term growth, though it is questionable whether increased AA and better neurodevelopment are directly related. Lipid infusion should be initiated right after birth, and further investigation will enable us to ascertain the potential impacts of lipid emulsions, particularly fish oil, on PI neurodevelopment. Conclusions: An aggressive PN and its possible metabolic complication could not favor neurodevelopment; the way forward could be a customized approach, depending on the patient’s clinical state and tolerance. Long-term follow-up studies and the search for specific markers of tolerance are warranted.

## 1. Introduction

The incidence of prematurity, defined as a birth occurring before 37 completed weeks of gestation, is increasing worldwide. It is estimated that approximately 12 to 15 million preterm births occur worldwide each year, with around 1 million of these infants dying due to complications related to prematurity; globally, prematurity is the leading cause of death in children under the age of 5 years [1,2].

Nevertheless, survival rates of preterm infants are increasing both in high- and lower-income countries because of advancements in prenatal and neonatal care and nutritional interventions soon after birth [3]. The rate of preterm birth ranges from 10–12% in low-and middle-income countries to 7% in high-income countries [2].

Postnatal malnutrition with extrauterine growth restriction (EUGR) remains a significant risk for the preterm population, particularly for infants born with a lower gestational age (GA) and birth weight, as they have limited nutritional reserves [4,5]. The incidence of EUGR is estimated to be up to 60–70% [6,7].

Preterm birth interrupts the physiological growth of the fetus occurring during the third trimester of pregnancy; as a result, early parenteral nutrition (PN) is needed to satisfy the increased metabolic demands of the premature newborn in order to minimize weight loss, initiate correction of intrauterine growth restriction (IUGR) and inadequate fetal nutrient storage, and prevent EUGR [3,8]. The immaturity of organ systems in preterm infants impacts a broad range of functions. The complex mechanisms behind preterm birth, including inflammation and cytokine damage, contribute to the development of bronchopulmonary dysplasia (BPD), necrotizing enterocolitis, brain white matter injury, and retinopathy of prematurity (ROP) [9].

Furthermore, preterm infants are at increased risk of neurodevelopmental impairment in terms of neuromotor disabilities, learning difficulties, and minor behavioral problems compared with infants born at term [10]. Some developmental processes, such as brain development during critical periods of organ growth, can be permanently altered by inadequate nutritional support. In order to meet the high metabolic demands of neurological tissues, it is essential to provide sufficient nutrients [11,12,13]. For example, processes such as myelination, which rises around 32 weeks of gestation, rely on adequate nutritional intake [14]. It is known that adequate amounts of protein and calories regulate the release of insulin-growth factor (IGF-1), which is crucial for growth and retinal vascularization [15]. Accordingly, poor postnatal weight gain in the first 4 weeks of life was found as a predictor of severe ROP, while adequate nutrition of preterm significantly reduced the incidence of ROP [16,17].

With improved survival rates, these outcomes have gained increased relevance. In several studies it has been demonstrated how improving early nutrition may favor the survival without neurodevelopmental impairment of preterm infants [11,12,13]. As a result, providing adequate nutritional care remains a significant challenge in the clinical practice.

For this reason, the aim of this systematic review was to assess the effect of early PN, in particular the provision of macronutrients, on neurodevelopmental outcomes of preterm infants in the first two years of life.

## 2. Materials and Methods

### 2.1. Search Methods for Identification of Studies

We conducted this systematic review in compliance with the Preferred Reporting Items for Systematic Reviews and Meta-analyses (PRISMA) guidelines [18]. The literature search was conducted up to May 2024 by two individuals independently on the Embase, MEDLINE, and Cochrane Library databases. The search strategy combined free-text terms related to both preterm infants’ PN and neurological outcomes in the first two years of life, as well as specific MeSH terms (“premature infants”, “parenteral nutrition”, “growth”, “brain”, “neurodevelopment”, and “central nervous system diseases”). Additional studies were identified through reference checking of all eligible articles. The search was not restricted by publication date, but it was limited to the English language.

To minimize selection bias, this review was conducted according to a predefined internal protocol that included a comprehensive search strategy (based on the PRISMA checklist [18]), strict eligibility criteria, and data extraction methods. There were no deviations from the original protocol.

### 2.2. Types of Studies, Participants and Outcomes Measures

Randomized controlled trials (RCTs), cohort studies, observational studies, and case-control studies were eligible for full-text review if they included very preterm infants (GA less than 32 weeks) and/or very low birth weight infants (birth weight less than 1500 g) receiving early PN during Neonatal Intensive Care Unit (NICU) stay. The outcomes considered were neurodevelopment in the first two years of life, measured by standardized neurodevelopmental scales (i.e., Bayley scale II or III, Griffith scale or others) and/or head circumference (HC) from 6 to 24 months of corrected age. Reviews, case series, notes and letters, conference abstracts, and opinion articles were excluded. Papers addressing both parenteral and enteral nutrition were also excluded if extraction of PN data was not possible. Studies in which the outcome indicators were assessed at different time points were also excluded.

### 2.3. Selection of Studies and Data Extraction

All retrieved records were imported into Covidence, an online collaboration software platform designed to streamline the process of producing systematic and other literature reviews [19]. Duplicates were removed automatically and manually. Three reviewers independently screened titles and abstracts for the study eligibility according to the above-mentioned criteria. The full texts of the selected references were then examined independently to confirm a study’s eligibility based on the inclusion and exclusion criteria. Any disagreement regarding study selection detected by Covidence was resolved by discussion between the researchers to reach a consensus.

For each selected article, two independent reviewers performed data extraction using a specifically built data collection form in Covidence. Extracted data were compared for any difference. If evidenced, differences were resolved by discussion and consensus between the researchers. For each selected study, the form summarized data on study characteristics (authors, year of publication, study design), population (GA, birth weight of patients enrolled in each treatment group for each study), characteristics of PN (standard or improved PN regimen with available macronutrients intakes), and outcomes (neurodevelopment using the Bayley scale or others standardized scales and/or HC).

### 2.4. Quality Assessment

Three reviewers independently rated the quality of the included studies. The Revised Cochrane Risk of Bias tool version 2 (RoB2) was used to assess the quality of all randomized clinical trials included in this review [20]. Cohort and case-control studies were assessed using the Newcastle–Ottawa Scale [21], while for cross-sectional studies, the Joanna Briggs Institute (JBI) Critical Appraisal Checklist was used [22]. The overall risk of bias judgments for each study did not affect their inclusion in this review.

### 2.5. Strategy for Data Synthesis

The review results were synthesized using qualitative methods. The qualitative synthesis took place through the characterization of the studies (table with the following information: authors, year of publication, objectives, design and methodology of the studies, and results obtained). The findings are presented in three main sections corresponding to key topics in parenteral micronutrient research in preterm infants: carbohydrates and energy, proteins, and lipids.

## 3. Results

### 3.1. Study Selection

**Figure 1:**
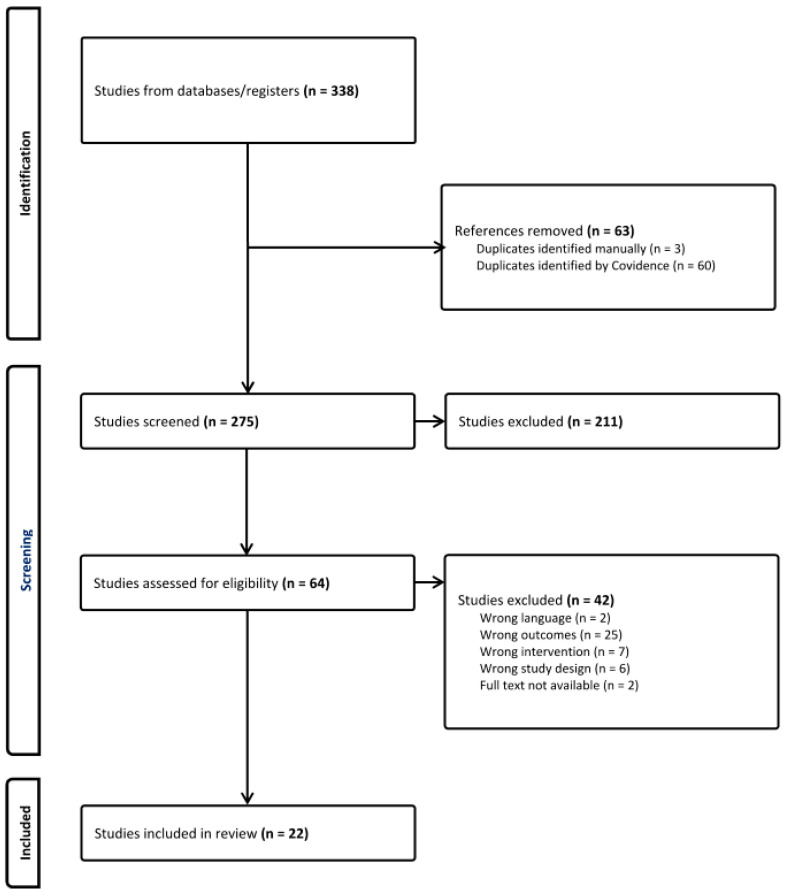
PRISMA diagram.

A total of 275 potential studies were retrieved from the databases and manual searches, excluding duplicates. After title and abstract screening, 64 studies were selected for full-text reading, and 22 of these met the eligibility criteria. The selection process is summarized in the PRISMA flow diagram to keep track of all papers included and the reasons for exclusion (Figure 1).

### 3.2. Study Characteristics

The final qualitative synthesis of the 22 studies included is detailed in Table 1. Each study was subsequently categorized based on its primary area of intervention: seven studies focused on energy and carbohydrate intake (Table 1a) [23,24,25,26,27,28,29], eight studies addressed protein intake (Table 1b) [30,31,32,33,34,35,36,37], and seven studies investigated lipid intake (Table 1c) [38,39,40,41,42,43,44].

### 3.3. Quality Assessment (Risk of Bias)

**Figure 2:**
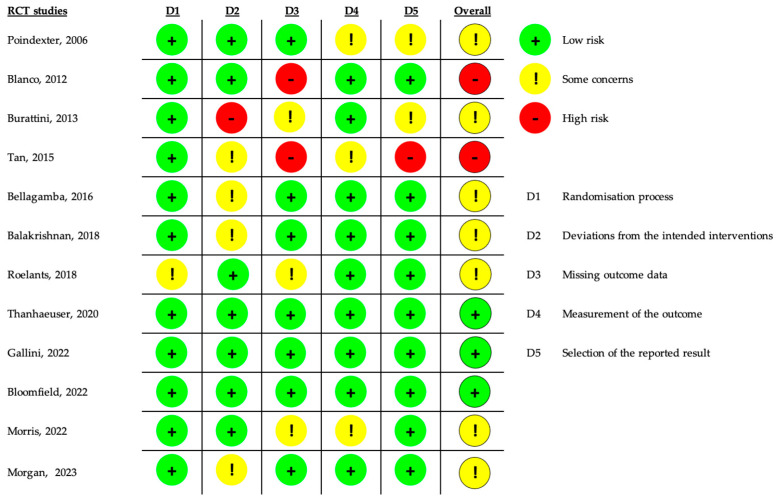
Summary of the Risk of bias assessment for the included RCT studies. Pointdexter [30], Blanco [31], Burattini [32], Tan [23], Bellagamba [35], Balakrishnan [36], Roelants [38], Thanhaeuser [41], Gallini [44], Bloomfield [37], Morris [28], Morgan [29].

The risk of bias assessment for randomized controlled trials is presented in Figure 2. All studies were evaluated for selection, performance, detection, attrition, and reporting biases using the RoB2 tool version 2019 [20]. The majority of studies (7 out of 12) were judged to have some concerns, while two studies were deemed to have an overall high risk of bias. Conversely, only three studies were evaluated as having an overall low risk of bias.

**Figure 3:**
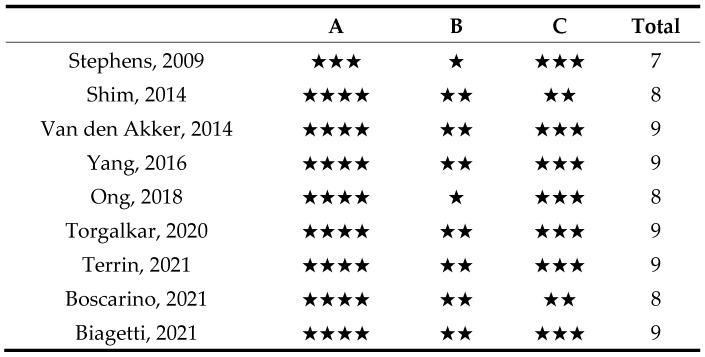
Summary of the Risk of bias assessment for the included cohort studies and case-control studies. A. Selection (maximum of four stars), B. Comparability (maximum of two stars), C. Outcome (maximum of three stars). Good quality: 3 or 4 stars in selection domain and 1 or 2 stars in comparability domain and 2 or 3 stars in outcome/exposure domain Fair quality: 2 stars in selection domain and 1 or 2 stars in comparability domain and 2 or 3 stars in outcome/exposure domain Poor quality: 0 or 1 star in selection domain OR 0 stars in comparability domain OR 0 or 1 stars in outcome/exposure domain. Stephens [24], Shim [25], Van den Akker [33], Yang [34], Ong [39], Torgalkar [40], Terrin [26], Boscarino [27], Biagetti [43].

The Newcastle–Ottawa scale (NOS) was used to evaluate the risk of bias in all included cohort studies (both prospective and retrospective) and case-control studies [21]. Selection bias, comparability bias, and exposure bias were evaluated for each study, as illustrated in Figure 3. Almost all studies were determined to have a low risk of bias.

The study selection included only one cross-sectional study conducted by Chen et al. [42]. The risk of bias assessment for this study was judged to be low following the Joanna Briggs Institute (JBI) Critical Appraisal Checklist [22].

## *4.* Studies Analysis

### 4.1. Carbohydrates and Energy

The cohort study by Shim et al. examined the effects of advanced energy intake through increased protein, lipid, and glucose (with a cumulative energy intake of 84.3 kcal/kg/day, which is 20 kcal/kg/day more than the placebo group) compared with a conventional nutritional regimen on determining language development assessed by the Denver Developmental Screening Test (DDST). In the intervention group, patients exhibited a higher rate of normal language development; in fact, only 2.8% of them exhibited language delay compared with 18.4% in the control group (*p*-value = 0.033); however, no significant effects were observed on other developmental categories or the diagnosis of cerebral palsy. Moreover, more aggressive protein intake (2.91 g/kg/day in the first group, higher than 1.53 g/kg/day in the control group) was associated with a significantly lower rate of postnatal growth restriction at 40 weeks corrected age, though no correlation was found with HC growth [25].

Similarly, Stephens et al. observed that higher energy intakes (about 60 ± 8 kcal/kg per day) and protein intakes (gradually increased up to 3.5 g/kg per day) mostly given by PN during the first week of life were significantly associated with better cognitive function at 18 months of corrected age. Higher Mental Development Index (MDI) scores on the Bayley scale emerged both for greater protein intake (8.2-point increase in MDI for each additional gram of protein per kilogram per day) and for higher energy intake (4.6-point increase in MDI for every 10 kcal/kg/day of energy intake). No significant associations were found between early nutrition and HC growth or psychomotor development [24].

Tan et al. examined the correlation between early nutrition and neurodevelopmental outcomes in extremely preterm infants. The present study focused on how improving nutritional intake with a PN containing 20% more energy (117 kcal/kg/day) during the early weeks of life might influence brain growth, measured by HC and magnetic resonance imaging (MRI), as well as developmental milestones during the first year. No significant benefits of enhanced energy intake were found on neurodevelopmental outcomes measured by the Bayley Scales of Infant Development (BSID-II) during the first nine months of life; however, there was a correlation between the energy deficit at 28 days of age and the total brain volume (TBV) at 40 weeks’ GA and MDI and PDI at 3 months post-term [23].

The randomized controlled trial by Morgan et al. sought to investigate the consequences of an enhanced nutritional regimen on the neurological development of very preterm infants. Newborns who received a concentrated, protein- and energy-enriched PN regimen (SCAMP) from the fourth day of life (76 vs. 64 kcal/kg/day in the intervention and control group, respectively, and protein amounts of 2.82 vs. 2.26 g/kg/day in the intervention and control group, respectively) exhibited superior cognitive, motor and linguistic abilities at 30 months corrected age compared with those who received standard nutrition, although these results were not statistically significant. In particular, the study showed higher motor (79 vs. 76, *p* = 0.38), cognitive (87 vs. 81, *p* = 0.08), language (81 vs. 76, *p* = 0.11), and combined composite score (84 vs. 78, *p* = 0.09) in the intervention group compared with the control group. Interestingly, the improvement in neurodevelopmental outcomes appears greater in infants with lower GA even though it did not reach statistical significance (mean difference between 5 and 10 in all the composite scores). The authors emphasize that, despite the increased caloric and protein intake, no rise in complications (hyperglycemia or electrolyte imbalances) was observed in the intervention group [29].

Morris et al. also aimed to determine whether increasing the intake of calories by administering higher glucose and lipid infusions early in life would improve motor and cognitive skills later on. No differences were observed in the Bayley Scales of Infant Development (BSID) at 12- and 24-months corrected age between the two groups of preterm infants, one treated with higher-caloric PN (mean parenteral intake of 81.6 kcal/kg/day) and the other with standard parenteral PN [28]. An association between early enhanced energy intakes and neurodevelopmental delay has been demonstrated in two studies. In particular, Boscarino et al. showed how increased energy intakes (energy intake through PN ≥ 432 kcal/kg/week) can lead to hyperglycemia (defined as two successive blood glucose concentrations that exceed 180 mg/dL at an interval of three hours), which negatively impacts neurodevelopment at 24 months of corrected age. Indeed, the rate of neurodevelopmental delay in preterm infants was found to be considerably elevated in the group that received a higher intake of all macronutrients in comparison with the control group [27].

The cohort study recently conducted by Terrin et al. suggests that higher energy intake early in life may not necessarily lead to improved long-term neurodevelopment in very low birth weight (VLBW) infants, and could potentially have adverse effects. Specifically, the study made a comparison between two cohorts of preterm infants. The first group received an energy-enhanced PN regimen, with an energy intake of 534.1 kcal/kg/week, while the second group was given a standard energy regimen of 411 kcal/kg/week during the first seven days of life. The nutritional protocols differed in total energy provided while maintaining the same protein intake. According to the Bayley Scales of Infant Development at 24 months of age, the results revealed that infants who received a greater provision of energy showed slightly worse motor skills and socio-emotional competence compared with those on standard PN. Nevertheless, no substantial dissimilarities were detected in the cognitive outcomes of the two groups [26].

### 4.2. Proteins

In a multicenter randomized controlled trial conducted in 2022, Bloomfield et al. examined an enhanced protein regimen (extra amino acids (AA) at a dosage of 1 g per day) supplied for the initial 5 days following birth in a population of extremely low birth weight (ELBW) compared with the group with standard nutrition (mean parenteral AA intakes of 2.6 ± 0.6 g per kilogram per day). The findings revealed no significant difference in survival without neurological impairment at a corrected age of 2 years (47.8% vs. 49.8% in the intervention and control group, respectively), as measured by the Bayley scales III; however, when evaluating secondary outcomes, these data indicated a potential rise in moderate-to-severe neurodisability among infants who received the intervention (27 out of 164 infants evaluated [16.5%] vs. 14 out of 163 [8.6%] in the placebo group, adjusted relative risk of 1.95; 95% CI, 1.09 to 3.48) [37].

Another randomized trial involving 168 participants found no difference in neurodevelopmental outcomes at 18–24 months corrected age between infants with a birth weight of 1250 g who received up to 4 g of AA per kilogram per day starting on the first day of life and those who followed a standard regimen of 2 g per kilogram per day. No differences were observed between the groups in any of the Bayley III components, nor in the rates of cerebral palsy, deafness, or blindness. Furthermore, after the exclusion of those who were SGA at birth, infants in the high AA group had lower HC z scores at discharge [36]. These findings support that starting AA infusions in preterm infants at the target intake levels from the first day of life, rather than gradually, is associated with no better outcomes. Similarly, Burattini et al. noted no differences in Bayley III scores at a 2-year follow-up in a cohort of 131 preterm infants with a birth weight of less than 1250 g, who were randomized to receive AA starting at birth with target doses of 2.5 g/kg/day versus 4 g/kg/day, accomplished by days 3–4. Both groups began with doses below their target levels, which were gradually increased over time to different final dose levels. Although infants in the higher AA group had, on average, 3 additional Bayley III points, more infants in this group (3 vs. 1) were removed because of severe mental retardation. Nonetheless, the authors outlined that a 3-point difference in Bayley would roughly need 1000 enrolled patients to reach a statistical significance considering the specific standard deviation and standardized difference [32].

In a large randomized controlled trial subjected to secondary analysis, Poindexter et al. demonstrated that early amino acids administration led to improved head growth at 36 weeks corrected age and a reduced number of infants with inadequate head development at 18 months. Nevertheless, no appreciable variations in neurodevelopmental outcomes were detected [30].

In another randomized trial from 2016, in 164 preterm infants with a birth weight below 1250 g, protein intake was increased by approximately 1 g/kg/day during PN and later during enteral nutrition, starting from the first day of life. In both groups, intravenous AA supplementation began at 1.5 g/kg/day and was increased to a maximum of 2.5 g/kg/day on day three for the standard group and 3.5 g/kg/day on day five for the high-protein group. The Bayley III scores showed no improvement in the intervention group at two years of follow-up [35].

Blanco et al. found that ELBW infants randomly assigned to a larger intravenous AA intake during the first week of life had significantly lower MDI scores at 18 months (starting at 2 g/kg/day at enrollment and increasing by 1 g/kg/day to a maximum of 4 g/kg/day), although, at the age of two, this difference was no longer significant; however, weight, length, and HC SDSs were much lower in the high AA group compared with the standard AA group. These findings should be interpreted cautiously, as this study was initially designed to investigate hyperkaliemia prevention and lacked the capacity to assess neurodevelopment [31].

Yang et al. detected a significant positive correlation between neurodevelopmental outcomes at age two and the amount of AA administered in the first few weeks of life. This small cohort study (46 ELBW infants were studied with Bayley III at 2 years of age) involved gradually increasing parenteral protein intake by 0.5–1.0 g/kg/day up to a maximum of 3.5–4 g/kg/day. A two-point improvement in language and motor scores on the Bayley III was associated with a higher parenteral AA intake during the second week; for every additional 1 g/kg/day of AA administered, both scores improved; however, the total AA intake during the second week was notably low: the median week 2 intake of parenteral AA was 2.5 g/kg/day and of total AA was 2.8 g/kg/day. As a result, the impact of increasing protein intake within the recommended ranges may have been overestimated [34].

Stephens et al. reported, in a cohort of 124 ELBW newborns, that a higher amount of calories and AAs during the first week of life was associated with better long-term growth and neurodevelopmental outcomes at 18 months. Specifically, increases of 4.6 and 8.2 points, respectively, in the Bayley II MDI were linked to an extra 10 kcal/kg of caloric and 1 g/kg of protein intake per day; however, the average protein intake during the first week was only 1.8 g/kg per day [24].

Long-term outcomes were reported by Van den Akker et al. in VLBW infants who had previously taken part in a randomized controlled trial that showed the short-term safety and effectiveness of 2.4 g/kg/day of AA from the first day of life. Notably, their results showed a sex difference in MDI scores, with boys achieving significantly better outcomes at the corrected age of 2 years; however, the data were collected retrospectively from small sample size and with potential selection bias (MDI was measured in only 71% of the girls with normal outcomes in the intervention group, compared with 84% in the control group) [33].

### 4.3. Lipids

#### 4.3.1. Amount of Lipids

Regarding the impact of varying intravenous lipid amounts during the initial days of life, Ong et al.’s follow-up study examined the effects of different intravenous soybean oil (SO) doses on neurodevelopment and growth in newborns <29 weeks of GA. Newborns from the original RCT were randomized to build up lipids intake in a low dose of SO (target 1.0 ± 0.2 g/kg/day) or a standard dose (target 2.6 ± 0.2 g/kg/day) of SO. At 6-, 12-, and 24-months corrected age, there were no significant differences between the two groups’ neurodevelopmental outcomes as measured by the Bayley Scales of Infant Development III. This study, however, was limited by the small sample size (30 patients completed the follow-up) [39].

Roelants et al. reported on the 2-year follow-up of a previous RCT the impact of administering mixed fat emulsion and high-dose of intravenously AA to VLBW newborns within the first two days of life. The intervention group [AA 3.6 g/kg/day, Smoflipid 2 g/kg/day (day 1)–3 g/kg/day(day 2)] did not have a beneficial effect on the incidence of major disabilities and neurodevelopmental outcomes (BSID-III) at 2 years of corrected age compared with the standard [AA 2.4 g/kg/day, Intralipid 2 g/kg/day (day 1)–3 g/kg/day (day 2)] and control [AA 2.4 g/kg/day and Intralipid 1.4 g/kg/day (day 1)–2.8 g/kg/day (day 2)] groups; however, enhanced HC growth was observed. The authors suggest that the limited time of intervention and the heterogeneity of the research groups may have contributed to the lack of a neurodevelopmental effect [38].

#### 4.3.2. Type of Lipids

Thanhaeuser et al. aimed to investigate whether the use of a mixed lipid emulsion containing fish oil could improve the neurodevelopmental outcome. ELBW neonates were randomized to receive either a mixed lipid emulsion containing fish oil (Smoflipid 20%) or a soybean oil-based lipid emulsion (Intralipid 20%). The neurodevelopment assessed at 12 and 24 months of corrected age using the Bayley Scales of Infant and Toddler Development (Bayley-III) showed no significant differences. The benefit was absent even in babies born <750 g, a subgroup in which docosahexaenoic acid (DHA) intake is known to be pivotal for brain development during pregnancy. The authors, however, emphasize that the absence of a beneficial effect could be due to the fact that the DHA supply via mixed lipid emulsion (43 mg/kg/d) was in the lower range of known accretion rates (40–67 mg/kg/day) [41]. Furthermore, the neurodevelopmental assessment in this study was a secondary outcome, so a lack of power for this study to detect significant differences in outcomes could be speculated.

These findings align with the results of a retrospective cohort study from Biagetti et al., which showed no difference in neurodevelopmental outcomes and the prevalence of neurodevelopmental delay at two years of corrected age between preterm babies receiving IV fish oil (IV-FO) or standard lipid emulsion [43].

A retrospective cohort study by Torgalkar et al. comparing Smoflipid and Intralipid’s effects on the neurodevelopmental outcomes in preterm infants born at less than 29 weeks GA found that Bayley III language scores of less than 70 and less than 85 at 18 to 24 months corrected age were lower in infants in the Smoflipid emulsion group compared with the Intralipid group [40].

However, since this study analyzes the outcomes of two populations from different epochs, it is not possible to rule out the possibility that advancements in newborn care throughout time may have contributed to the improvements in outcomes.

Gallini et al. assessed the neurodevelopmental outcomes of babies enrolled in an RCT from Costa et al. [45] at 24 months corrected age using the Griffiths Developmental Scale. No differences between the two groups were evidenced. These studies suggest that while fish oil-containing lipid emulsions may show initial benefits in head growth, they may not necessarily correlate with improved neurodevelopmental outcomes [44]. According to Chen et al., children who previously received lipid emulsions with fish oil instead of a standard soybean oil-based emulsion had a lower incidence of epilepsy, cerebral palsy, developmental disorder, and attention-deficit-hyperactivity disorder (ADHD) at two years of corrected age [42]. The results, however, are difficult to compare with Gallini’s [44] since, as the author suggests, Chen et al.’s study was retrospective cross-sectional rather than RCT. In addition, the mean GA of the infants enrolled in Chen et al.’s study was higher (mean of 32.2 weeks GA) than that of Gallini’s (mean of 28.1 weeks GA), and also they administered a different parental lipid emulsion (Lipofundin instead of Intralipid).

## 5. Discussion

### 5.1. Energy and Carbohydrates

All macronutrients (lipids, proteins, and carbohydrates) contribute to determining caloric intake. In accordance with the latest guidelines, in order to ensure adequate energy requirement when using PN, it is recommended to start a minimum of 45–55 kcal/kg/day and to gradually increase the intake to 90–120 kcal/kg/day in the maintenance phase. The recommended glucose intake for preterm infants is 4–8 mg/kg/min (5.8–11.5 g/kg/day) on the first day of life, with a gradual increase to the target dose of 8–10 mg/kg/min (11.5–14.4 g/kg/day), with this adjustment made in consideration of the infant’s illness phase (i.e., acute, stable, recovery/growth) [46,47] (Table 2).

However, the optimal provision of calories for preterm infants remains to be elucidated, and as such, an individualized determination of the need for micro and macronutrients should be performed based on each patient, taking into account both their clinical condition and individual metabolic capacity. The balance between meeting energy needs and the risks of overfeeding/excess glucose load leading to hyperglycemia, the phase of illness, and glucose administered outside PN should all be taken into consideration when determining the amount of glucose to be provided by PN [46,47].

Some of the literature findings support that optimizing first-week energy intakes could have important implications for improving long-term neurodevelopmental outcomes in very preterm infants. Nevertheless, accurate monitoring of the individual patient’s metabolic response to macronutrient intake is strongly recommended in order to exclude potential adverse effects that could impact neurological outcomes.

### 5.2. Proteins

Current recommendations suggest that, in order to foster an anabolic condition, preterm newborns should start AA intake on the first postnatal day at a rate of at least 1.5 g/kg/day. From the second day onward, parenteral AA intake should fall between 2.5 and 3.5 g/kg/day, in addition to adequate micronutrient intakes and non-protein calories exceeding 65 kcal/kg/day [48]. Overall, early AA supplementation is linked to improved short-term growth, but how it affects long-term outcomes such as growth and neurodevelopment is less understood. Furthermore, since there is no agreement on a normative AA profile, it is still unclear how much protein is too much.

Inadequate protein intake during the neonatal period can lead to energy deficiency, which has been directly linked to poor head growth [49]. Reduced head circumference has been associated with lower developmental indices and cognitive abilities in later life, particularly in infants born with very low birth weight (VLBW) [50,51].

However, the evidence supporting a direct link between higher AA intake and improved neurodevelopment is inconclusive. This aligns with the findings of a 2018 Cochrane meta-analysis, which examined parenteral AA intake in preterm infants and its impact on neurodisability and suggested no reported benefit [52] (Table 2). Since neurodevelopment in preterm infants is influenced by multiple factors, well-powered trials are needed to identify the optimal AA intake and the implications of the caloric balance of PN on brain development and neurodevelopmental outcomes.

### 5.3. Lipids

In preterm infants according to ESPGHAN guidelines, lipids infusion should be initiated right after birth and not later than the second day of life. There is no evidence that fat tolerance is enhanced by a slow increase in the infusion rate. Currently, the maximum amount of lipids intake in preterm infants is not known with certainty; however, an upper threshold of 4 g/kg/day has been demonstrated to be safe. Interestingly, the guidelines highlight that this threshold is lower than what would be provided with full enteral feeding [53] (Table 2).

The impact of fish oil-containing lipid emulsions on neurodevelopmental outcomes in ELBW neonates remains a controversial topic. Lipid composition may influence outcomes differently in extremely low birth infants depending not only on lipid emulsion composition but also on the DHA newborn’s status at birth, which seems to depend on the mother’s DHA synthesis [54]. While some studies have suggested potential benefits, others have failed to find significant differences between fish oil-containing and soybean oil-based lipid emulsions. Firstly, the dose of DHA provided in the studies is variable, and it is possible that lower doses (often in the lower range of known accretion rate) may not be sufficient to exert a significant impact on neurodevelopment. Secondly, the timing of DHA supplementation may be delayed.

The methodological limitations of some studies, such as the retrospective design or the small sample sizes, could make it difficult to draw definitive conclusions. Thus, large-scale randomized controlled trials to identify differences in neurodevelopment would be beneficial.

**Table 2:** Current recommendations in accordance with the established guidelines, neurological outcomes derived from the extant literature, and future needs.

	Energy	Carbohydrates	Proteins	Lipids
Recommended Intakes	[46]	[47]	[48]	[53]
Start (1st day)	At least 45–55 kcal/kg/d	5.8–11.5 g/kg/d	At least 1.5 g/kg/d	<2–3 g/kg/d
Maintenance	90–120 kcal/kg/d	11.5–14.4 g/kg/d	2.5–3.5 g/kg/d from day 2* * with non-protein intakes exceeding 65 kcal/kg/d and sufficient micronutrients	Upper threshold of 4 g/kg/d
Literature Review				
Neurological Outcome	Optimizing first-week energy intake could have implications for long-term neurodevelopment (mainly observational studies)	Tailored glucose maintenance should be provided to avoid hypo/hyperglycemia	Promoting an anabolic state with immediate AA provision. The relationship between high levels of AA intake and improved neurodevelopment is uncertain	Lipid infusion should be initiated right after birth. The possible impact of fish oil-containing lipid emulsion on neurodevelopment needs to be further assessed
Future Needs				
	A feasible method to know the energy expenditure of preterm newborn	Bedside method for real-time glucose monitoring	Biochemical markers for anabolic state and AA tolerance	Biochemical markers to detect the best amount of lipid intake to be provided. Tailored provision for LC-PUFA

Table 2 provides an overview of the current ESPGHAN guideline recommendations on nutritional intakes for preterm infants [46,47,48,53]. It also highlights the findings from this literature review regarding the impact of macronutrient intakes on neurological outcomes and emphasizes the need for further research to address existing gaps in knowledge.

## 6. Conclusions

In conclusion optimization of nutritional care through adequate parenteral macronutrient intakes immediately afterbirth is mandatory and may positively impact neurodevelopmental outcomes. In recent years, many steps forward have been taken on this topic, and specific guidelines for this special population are available; however, it is reported that some aspects, such as optimal energy, protein, and lipid intakes, should be further assessed and individualized. Feasible devices measuring energy expenditure and real-time glycemia in preterm, together with the study of markers of amino acids tolerance and lipid status, could help us optimize macronutrient intakes. We have learned that an aggressive approach to nutrition could lead to severe metabolic complications and may not correlate with favorable outcomes and should be replaced with a customized one, depending on the state and tolerance of the patient. Future research, comprehensive of long term follow up, will help us to understand the potential benefit of early nutrition further.

## Figures and Tables

**Table 1:** Final qualitative synthesis of the considered studies 1a. Energy and carbohydrates. Legend for Table 1a–c: RCT ran-domized-controlled trial, GA gestational age, WK weeks, SGA small for gestational age, AGA appropriate for gestational age, G grams, I intervention group, C control group, MAX maximum, MO months, MDI Mental Development Index, PDI psycho-motor development index, PN parenteral nutrition, SD standard deviation, SDS standard deviation score, N number, CA corrected age, AA amino acids, NDI neurodevelopmental impairment, ND neurodevelopment, VS versus, UAC umbilical arterial catheter. (b) Proteins (c) Lipids.

**(a)**								
**Energy and Carbohydrates**	**Population**	**Group Differences**	**Birth Weight (g)**	**Mean Age (GA)**	**Parenteral Intakes**	**Neurological Outcome**		**Conclusions**
Tan, 2008 RCT [23]	GA < 29 wk	Enhanced Nutrition Protocol VS Standard parenteral nutrition protocol (during 1st month of life)	I 915 C 980	I 27 (25–28) C 27 (26–28)	Energy intake (median, IQ range): I 2762 kcal/kg (2438 to 3088); C 2654 kcal/kg (2395 to 2914) Protein intake (median, IQ range); I 74.8 g/kg (61.7 to 87.9); C 64.2 g/kg (58.7 to 69.7)	9 mo CA	MDI (mean, SD): I 91 (72–110); C 91 (73–109) PDI (mean, SD): I 90 (67–113); C 88 (69–107) Head circumference (mean, range): I 44.6 (42.6–46.6); C 45.4 (42.4–48.4)	Mental development index (MDI) and psychomotor development index (PDI) were not statistically different between the two groups
Stephens, 2009 Retrospective cohort study [24]	BW < 1000 g GA < 32 wk	Single cohort of patients receiving parenteral nutrition according to the same nutritional guidelines (during the first 7 days of life)	787 (SGA 13%)	25.9	Energy intake (kcal/kg/d): 60 (week 1) to max 105 (week 3–4) Protein intake (g/kg/d): 1.8 (week 1) to 3.3–3.5 (week 2–4)	18 mo CA	MDI (mean, SD): 79 ± 16 PDI (mean, SD): 78 ± 15 Head circumference (mean, SD): 47.1 ± 1.8	Increased first-week protein and energy intakes are associated with higher Mental Development Index scores at 18 months in extremely low birth weight infants
Shim, 2014 Prospective cohort study [25]	BW < 1500 g	Enhanced Nutrition Protocol VS Standard parenteral nutrition protocol (during 1st month of life)	I 1170 ± 200 (SGA 15.4%) C 1210 ± 190 (SGA 10.5%)	I 29 + 2 C 29 + 3	Energy intake (kcal/kg/d): I 84.3; C 63 Protein intake (g/kg/d): I 3 to 3.5–4; C 0.5 to 3 Lipid intake (g/kg/d): I 2 to 3.5–4; C 0.5 to 3	18 mo CA	Denver Developmental Screening Test II (delay n, %) - Language delay: I 2 (3.8); C 7 (18.4) - Gross motor delay: I 7 (13.5); C 6 (15.8) - Fine motor delay: I 2 (3.8); C 1 (2.6) - Personal-social delay: I 4 (7.7); C 1 (2.6) Cerebral palsy (n, %): I 5 (9.6); C 4 (10.5)	The early aggressive nutrition group had higher rates of normal language development at 18 months corrected age compared with the conventional nutrition group
Terrin, 2021 Prospective cohort study [26]	BW < 1500 g GA < 32 wk	Two PN protocols with different energy intakes but the same protein intake (during the first 7 days of life)	I 1214 (SGA 29.2%) C 1215 (SGA 22.2%)	I 29 (28–30) C 29 (28–30)	Carbohydrate intake (g/kg/d): I (<1000 g) 7 to 16, (>1000 g) 8.5 to 15; C (<1000 g) 7 to 14, (>1000 g) 7 to 14.5 Energy intake (kcal/kg/d): I (<1000 g) 55 to 120, (>1000 g) 60 to 110; C (<1000 g) 45 to 105, (>1000 g) 45 to 100 Protein intake (g/kg/d): I (<1000 g) 2 to 4, (>1000 g) 2 to 3.5; C (<1000 g) 2 to 4, (>1000 g) 2 to 3.5 Lipid intake (g/kg/d): I (<1000 g) 2 to 4, (>1000 g) 2 to 3.5; C (<1000 g) 1 to 3.5, (>1000 g) 1 to 3	24 mo CA	Bayley III (mean, SD) - Language composite score (I 86 ± 14; C 84.5 ± 12.5) - Motor composite score (I 91 ± 12; C 97 ± 15) - Cognitive composite score (I 90 ± 15; C 87.5 ± 6.2) Head circumference (z-score, IQR): I −0.2 (2.3); C −0.4 (1.4)	A more aggressive PN strategy results in lower motor score and socioemotional competence performance at 24 mo of life
Boscarino, 2021 Case-control study [27]	BW < 1500 g GA < 32 wk	Exposed to hyperglycemia VS Not exposed to hyperglycemia (during the first 12 days of life)	I 1010 (SGA 20%) C 1347 (SGA 22%)	I 27 C 30	Energy intake: I higher; C lower	24 mo CA	Bayley III (mean, SD) - Language composite score (I 76; C 82.5) - Motor composite score (I 84.5; C 94.3) - Cognitive composite score (I 83; C 88.9)	High nutritional intakes through PN soon after birth increase the risk of hyperglycemia, which affects long-term neurodevelopment (higher rate of cognitive and motor delay) and survival in preterm newborns
Morris, 2022 RCT [28]	BW < 1500 g GA < 32 wk	Enhanced Nutrition Protocol VS Standard parenteral nutrition protocol (during first 8 days of life)	I 900 (SGA 3.9%) C 1000 (SGA 4.9%)	I 27 C 27.4	Carbohydrate intake (mg/kg/min): I 5.5; C 1 to 12–14 Energy intake (kcal/kg/d): I 81.6 (SD 11.1); C 65.2 (SD 10.5) Protein intake (g/kg/d): I 4; C 3 to 4 Lipid intake (g/kg/d): I 2 to 3.5; C 0.5–1 to 3.5	24 mo CA	Bayley III (mean, SD) - Language composite score (I 95.5 ± 18.2; C 94.8 ± 22.1) - Motor composite score (I 88.1 ± 19.9; C 89.2 ± 20.4) - Cognitive composite score (I 96.3 ± 14.9; C 92.3 ± 22.8)	Enhanced early PN was not associated with improved neurodevelopment
Morgan, 2023 RCT [29]	BW < 1200 g GA < 29 wk	Enhanced nutrition protocol (SCAMP) VS Standard parenteral nutrition protocol (during first 14 days of life)	I 900 C 884	I 26.8 C 26.6	Energy intake (kcal/kg/d): I 76 (SD 14); C 64 (SD 8) Protein intake (g/kg/d): I 2.82 (SD 0.51); C 2.26 (SD 0.42)	24 mo CA	Bayley III (mean, SD) - Language composite score: I 81 (18); C 76 (17) - Motor composite score: I 79 (13); C 76 (15) - Cognitive composite score: I 87 (15); C 81 (14) - Combined composite score: I 84 (15); C 78 (14)	No significant improvement in ND outcome when administering higher parenteral protein/energy intakes
**(b)**								
**Proteins**	**Population**	**Group Differences**	**Birth Weight (g)**	**Mean Age (GA)**	**Parenteral Intakes**	**Neurological Outcome**		**Conclusions**
Poindexter, 2006 RCT [30]	BW 401–1000 g AGA	Infants were stratified by whether they were provided >3 g/kg/d of AA at <5 days of life or not	I 805 ± 127 C 791 ± 127	I 26.1 ± 1.5 C 26 ± 1.5	Energy intake (kcal/kg/d): I 43.3 ± 13.9; C 31.2 ± 11.4 Protein intake (g/kg/d): I ≥ 3; C < 3	18–24 mo CA	Head circumference (mean, SD): I 47.1 ± 1.9; C 46.2 ± 2 Cerebral palsy (n, %): I 17 (26); C 99 (14) MDI (mean, SD): I 78.1 ± 16.1; C 79 ± 18.2	At 18 months’ CA, there were no differences in weight, length, or measures of neurodevelopment between the groups; however, male infants in the late group were twice as likely to have head circumference less than the 10th percentile.
Blanco, 2012 RCT [31].	BW < 1000 g GA > 24 wk	IV-AA starting at 0.5 g/kg/d and increased by 0.5 g/kg every day to 3 g/kg/d or starting at 2 g/kg/d and advanced by 1 g/kg every day to 4 g/kg/d	I 820 C 805	I 26.5 C 26.3	Protein intake (g/kg/d): I 2 to 4; C 0.5 to 3	18–24 mo CA	Cerebral palsy (n): I 3; C 1 MDI (mean, SD): I 57 ± 11; C 63 ± 13	The early and high AA group had a lower MDI at 18 months. This difference disappeared at 2 years of age.
Burattini, 2013 RCT [32].	BW 500–1249 g	Preterm infants were randomized to receive amino acids starting at birth with target doses of 2.5 g/kg/day versus 4 g/kg/day, achieved by days 3–4	I 974 ± 182 (SGA 11%) C 994 ± 194 (SGA 12%)	I 201 d ± 14 C 201 d ± 15	Carbohydrate intake (g/kg/d): 6–12 Protein intake (g/kg/d): I 2.5 to a max of 4 on day 4; C 1.5 to a max of 2.5 on day 3 Lipid intake (g/kg/d): 0.5–2.5	24 mo CA	Bayley III (mean, SD) - Combined composite score: I 97 ± 15; C 94 ± 13 Head circumference (mean, SD): I 48.1 ± 1.9; C 48.4 ± 1.6	No difference in the Bayley III score at the 2-year follow-up.
Van den Akker, 2014 RCT [33]	BW < 1500 g GA < 32 wk	Infants were randomized to receive the standard nutritional protocol or an early amino acid protocol. The intervention group received glucose and 2.4 g/kg/day amino acids within 2 h following birth on the first 3 days. After day 3 of life, the infants in both groups were subjected to the same standard nutritional protocol	-	-	Carbohydrate intake (mg/kg/min): 5.5 D1; 5.6 D2; 5.7 D3; 7.1 D4 Protein intake (g/kg/d): I 2.4 D1–D4; C 0 D1, 1.4 D2–3, 4 D4 Lipid intake (g/kg/d): 0 D1, 1.4 D2–D3, 2.8 D4	18–24 mo CA	Head circumference (mean, SD): I 48.4 ± 1.9; C 48.2 ± 1.7 Cerebral palsy (n): I 0; C 3 MDI (mean, SD): I 93.1 ± 9.8; C 96.6 ± 12	Premature boys, but not girls, benefited from amino acid administration directly following birth.
Yang, 2016 Retrospective cohort study [34]	BW < 1250 g	The amount of amino acids and calories received in the first 4 weeks of life were collected in a single cohort of patients	948 (SGA 20%)	28 (24–31)	Protein intake (g/kg/d): 1.4 (0.6–2.1) wk 1; 2.5 (1.3–3.7) wk 2; 2.9 (0–3.7) wk 3; 2.6 (0–3.7) wk 4 Lipid intake (g/kg/d): 1.5–2 to 4 Energy intake (kcal/kg/d): target 110–130	24 mo CA	Bayley III (mean, SD) - Language composite score: 86 (47–118) - Motor composite score: 88 (61–130) - Cognitive composite score: 95 (70–120)	Amino acid intake within the first weeks of life correlated positively with neurodevelopmental outcomes at 2 years (higher language and motor scores on the 2-year Bayley Scales).
Bellagamba, 2016 RCT [35]	BW 500–1249 g	Study infants were randomized to: standard of care nutrition and protein intakes (StP, standard protein) the intervention group (HiP, high protein)	I 885 ± 150 (SGA 6%) C 906 ± 157 (SGA 7%)	I 27 (26–28) C 27 (26–29)	Carbohydrate intake (g/kg/d): 6–12 Protein intake (g/kg/d): 1.5 to 3.5 Lipid intake (g/kg/d): 1–2.5	18–24 mo CA	Head circumference (mean, SD): I 47.6 ± 2.34; C 47.9 ± 2.17 Cerebral palsy (n): I 1; C 2 MDI (mean, SD): I 94 ± 13.9; C 93.8 ± 12.9	Increasing AA/protein intake both during parenteral and enteral nutrition does not improve neurodevelopment at 2 years corrected age.
Balakrishnan, 2018 RCT [36]	BW 400–1250 g GA 24–30 + 6 wk	All infants were started on a standard hyperalimentation (HAL) solution containing dextrose, calcium, and 1–2 g/kg/d AA (depending on total volume). Then, infants were randomized to receive either “standard” or “high” AAs in their HAL. Total AA intake reached equivalence between groups on day 7	I 877 (SGA 20%) C 888 (SGA 12%)	I 26.9 (24–30) C 26.6 (24–30)	Protein intake (g/kg/d): I 3–4 and advancing to 4 by day 1; C 1–2 and advancing daily by 0.5 to a goal of 4	24 mo CA	Bayley III (mean, SD) - Language composite score: I 90.3 ± 16.2; C 88 ± 13.4 - Motor composite score: I 93 ± 13.7; C 93.1 ± 12.3 - Cognitive composite score: I 90.6 ± 12.7; C 90.2 ± 10.3 Cerebral palsy (n, %): I 4 (7); C 6 (10)	Current recommendations for high-dose AA starting at birth are not associated with improved growth or neurodevelopmental outcomes.
Bloomfield, 2022 RCT [37]	BW < 1000 g UAC in situ	Starting within 24 h after birth and continuing for 5 days, infants received: I: 8.5% TrophAmine (B Braun Medical) containing 1 g of amino acids and 1.4 mg of sodium in 12 mL of fluid C: 0.45% saline containing 21.2 mg of sodium in 12 mL of fluid	I 770 (SGA 10%) C 789 (SGA 12%)	I 25.4 C 26	Protein intake (g/kg/d): I 3.4 (±0.6 SD); C 2.6 (±0.6 SD)	24 mo CA	Bayley III (mean, SD) - Language composite score: I 89.4 ± 17.1; C 92.5 ± 16.5 - Motor composite score: I 94.7 ± 15.7; C 95.9 ± 13 - Cognitive composite score: I 94.2 ± 15.6; C 95.7 ± 14.4 Cerebral palsy (n, %): I 13 (8.1); C 9 (5.5)	Extra parenteral amino acids at a dose of 1 g per day for 5 days after birth did not increase the number who survived free from neurodisability at 2 years.
**(c)**								
**Lipids**	**Population**	**Group Differences**	**Birth Weight (g)**	**Mean Age (GA)**	**Parenteral Intakes**	**Neurological Outcome**		**Conclusions**
Roelants, 2018 Follow-up of RCT study [38]	BW < 1500 g	C glucose, standard-dose amino acids (AAs 2.4 g/kg/d) + pure soybean oil fat emulsion (SOY) (n = 46) I1 glucose, standard-dose AA+ SOY (n = 24) I2 glucose, standard-dose AA+ mixed fat emulsion (MIX, n = 25). I3 glucose, high-dose AAs (3.6 g/kg/d) + SOY (n = 24) I4 glucose, high-dose AAs (3.6 g/kg/d) + MIX (n = 23)	C 863 (SGA 4%) I1 808 (SGA 0%), I2 846 (SGA 0%), I3 775 (SGA 4%), I4 850 (SGA 5%)	C 27 + 3 I1 26 + 2 I2 27 + 1 I3 26 + 5 I4 27 + 1	Carbohydrate intake (g/kg/d): 6 Energy intake (kcal/kg/d): C 44.6–63.4; I1 62.6–71.6; I2 62.6–71.6, I3 67.4–76.4, I4 67.4–76.4 Protein intake (g/kg/d): C 2.4; I1 2.4, I2 2.4, I3 3.6, I4 3.6 Lipid intake (g/kg/d): C 1.4–2.8; I1 2–3, I2 2–3, I3 2–3, I4 2–3f	24 mo CA	Bayley III (median, IQR) - Mental score: C 100 (91–107); I1 100 (97–105), I2 97 (91–107), I3 97 (91–107), I4 104 (92–109) - Psychomotor score: C 98 (86–105); I1 100 (95–105), I2 100 (93–105), I3 95 (88–100), I4 100 (90–110) Head circumference (SDS, 95% CI): I1 −0.69 (−1.4 to 0.01), I2 −0.48 (−1.23 to 0.26), I3 −0.30 (−1 to 0.41), I4 0.61 (−0.07 to 1.28) Cerebral palsy (n, %). C 1 (2), I1 0 (0), I2 1 (4), I3 2 (8), I4 3 (14)	Neurodevelopmental scores and incidence of major disabilities did not differ between groups at 2 years corrected age (only the weight scores were higher in the high AA + MIX group)
Ong, 2018 Prospective cohort study [39]	GA ≤ 29 wk	Preterm neonates were randomized after birth to: the low-dose (LOW); standard dose (CON) of soybean oil	I 1033 ± 279 (SGA 11%) C 1023 ± 306 (SGA 16%)	I 28 ± 1 C 27 ± 1	Carbohydrate intake (g/kg/d): I 5.3 ± 0.8 (day 1) vs. 8 ± 0.8 (day 28): C 5.4 ± 0.8 (day 1) vs. 9.2 ± 0.8 (day 28) Energy intake (kcal/kg/d): I 32.5 ± 6.1 (day 1) vs. 28 ± 6.1 (day 28); C 33 ± 6.1 (day 1) vs. 32.8 ± 6.1 (day 28)	6–12–24 mo CA	Bayley III The frequency of Low Developmental Domain Scores < 1 SD below the normative mean at 24 mo (n, %) - Language composite score: I 2 (18); C 4 (29) - Motor composite score: I 2 (18); C 1 (7) - Cognitive composite score: I 3 (27); C 2 (14)	There were no differences in ND and growth outcomes when LOW was compared with CON, with the exception of a higher 12-month follow-up cognitive scaled score in the LOW group
Torgalkar, 2020 Retrospective cohort study [40]	GA < 29 wk at least 7 days of lipid emulsion	Group 1 (I) received soy-based LE (Intralipid 20%) Group 2 (C)received SMOF-LE (Smoflipid 20%)	I 890 (SGA 8%) C 879 (SGA 10%)	I 26 C 26	Lipid intake (g/kg/d): 1 (increased by 1 every 24 h) to a max of 3–3.5	18–24 mo CA	Bayley III (n, %) - Language score < 70: I 36 (13.3); C 9 (5.9) - Motor score < 70: I 16 (5.8); C 6 (3.7) - Cognitive score < 70: I 15 (5.3); C 7 (4) Cerebral palsy (n, %): I 20 (6.6); C 13 (6.9)	The composite outcome of death or significant NDI did not differ significantly. The odds of death or any NDI were lower in the SMOF-LE group
Thanhaeuser, 2020 RCT [41]	BW < 1000 g	Participants were randomized and stratified (sex and birth weight <750 g) to receive parenteral nutrition using: a mixed lipid emulsion (Smoflipid 20%; Fresenius Kabi, Bad Homburg, Germany; composed of 30% soybean oil, 30% medium chain triglycerides, 25% olive oil, and 15% fish oil; ω-6:ω-3 ratio 2.5:1); a soybean oil-based lipid emulsion (Intralipid 20%; Fresenius Kabi, Bad Homburg, Germany; ω-6: ω-3 ratio 8:1)	I 772.5 (SGA 20%) C 760 (SGA 31%)	I 25 + 5 (24 + 6 to 27 + 1) C 26 + 2 (25 + 0 to 28 + 0)	Lipid intake (g/kg/d): I 2 (1.6 to 2.2) and DHA 43 (35 to 48); C 1.9 (1.6 to 2.1) and DHA 3.8 (3.2 to 4.2)	24 mo CA	Bayley III (mean, SD) - Language composite score: I 89 (75–97); C 89 (77–100) - Motor composite score: I 94 (82–103); C 94 (85–103) - Cognitive composite score: I 95 (80–105); C 95 (90–105) Head circumference (mean, SD): I 47 (46–48.5); C 47 (46–48)	Parenteral nutrition using a mixed lipid emulsion containing fish oil did not improve the neurodevelopment of ELBW infants at 12- and 24-months corrected age
Chen, 2021 Retrospective cross-sectional study [42]	For premature babies, ICD-9 codes between 765.20–765.28	All patients who received Smoflipid or Lipofundin MCT/LCT were divided into an SMOF and a LIPO group, respectively. Patients in each group were further stratified by birth weight (≥1500 g and <1500 g subgroups)	I 1759.8 (SD 514.6) C 1764.9 (SD 558.7)	I 32.12 (SD 2.38) C 32.15 (SD 2.38)	Protein intake (g/kg/d): 3.5 Lipid intake (g/kg/d): 3	24 mo	Cerebral palsy in the subgroup <1500 g (number, %): I 6 (2); C 18 (6.1)	Lipid emulsions with fish oil improve the neurodevelopmental outcomes of children born prematurely
Biagetti, 2021 Case-control study [43]	BW 400–1249 g GA 24–35 wk	Two groups according to the LE received: the IV-FO group received MSF (50:40mediumchaintriglycerides-MCT: soybean oil, 10% fish oil-FO; Lipidem Braun) and MOSF (30:30:25 MCT:soybean oil:olive oil,15% FO; Smoflipid, Fresenius Kabi); the control group (CNTR) received S (100% soybean oil; Intralipid, Fresenius Kabi), MS (50% MCT and 50% soybean oil; Lipofundin MCT, B Braun) and OS (80% olive oil and 20% soybean oil; Clinoleic, Baxter)	I 931 ± 182 (SGA 26%) C 944 ± 194 (SGA 31%)	I 197 ± 14 days C 198 ± 15 days	Lipid intake (g/kg/d): I DHA 37 ± 6; C DHA 8 ± 1	24 mo CA	Bayley III (mean, SD) - Language composite score: I 93 ± 17; C 94 ± 17 - Motor composite score: I 98 ± 12; C 100 ± 13 - Cognitive composite score: I 92 ± 15; C 93 ± 13 Head circumference (mean, SD): I 48.1 ± 1.8; C 48 ± 1.8	No differences in neurodevelopment at 2 years corrected age
Gallini, 2022 RCT [44]	BW ≤ 1250 g GA ≤ 30 wk	Infants were randomized to receive: a multicomponent lipid emulsion (Smoflipid 20%, Fresenius Kabi, Germany; study group); the traditional soybean oil-based lipid emulsion (Intralipid 20%, Fresenius Kabi, Germany; control group)	I 865 ± 225 (SGA 40%) C 895 ± 220 (SGA 28%)	I 28 ± 2.5 C 28.2 ± 1.9	Energy intake (kcal/kg/d): I 61.6 ± 20; C 58.1 ± 23.5 Lipid intake (g/kg/d): 1.7 ± 0.6 (DHA 2%); 1.6 ± 0.7 (DHA 0%) Protein intake (g/kg/d): I 2.3 ± 0.8; C 2.2 ± 0.9	24 mo CA	Cerebral palsy (n, %): I 1 (2.7); C 2 (5) Head circumference (mean, SD): I 46.9 ± 2; C 47.3 ± 1.7	No significant effect on neurodevelopment outcomes at 24 mo of CA
